# Trauma and mental health in Pacific Islanders

**DOI:** 10.1177/00207640241236109

**Published:** 2024-03-15

**Authors:** Andrew M Subica, Lolofi Soakai, Amen Tukumoeatu, Taffy Johnson, Nia Aitaoto

**Affiliations:** 1 School of Medicine, University of California, Riverside, USA; 2 Motivating Action Leadership Opportunity, Pomona, CA, USA; 3 Empowering Pacific Islander Communities, Inc., Portland, OR, USA; 4 United Territories of Pacific Islanders Alliance, Kent, WA, USA; 5 Pacific Islander Center of Primary Care Excellence, San Leandro, CA, USA

**Keywords:** Native Hawaiian/Pacific Islanders, trauma, mental health, substance use

## Abstract

**Background:**

Little is known about trauma and its mental health impact on Native Hawaiians/Pacific Islanders (NH/PI), an understudied Indigenous-colonized population that endures severe mental health disparities.

**Aims:**

This novel investigation assessed trauma prevalence and its mental health and substance use correlates in NH/PIs in the U.S.

**Method:**

Using community-based participatory research methods, survey data on NH/PI trauma, depression, anxiety, substance use, and treatment need were collected from 306 NH/PI adults using online, telephone, and in-person methods. Descriptive statistics and adjusted regression models were employed.

**Results:**

Sixty-nine percent of participants experienced lifetime trauma, reporting mean exposure to 2.5 different trauma types. Childhood physical and sexual abuse, and lifetime forced sexual assault rates were 34%, 25%, and 27%, respectively, exceeding general population rates. Women and men reported equivalent total mean exposure to different trauma types, as well as equal prevalence for every trauma type examined (e.g. sexual abuse/assault). Confirming hypotheses, after controlling for key demographic and mental health risk factors, increased exposure to multiple trauma types uniquely associated with greater depression, anxiety, alcohol symptomology, and greater likelihood for needing treatment and using illicit substances.

**Conclusions:**

Trauma is prevalent in NH/PI populations and significantly impacts NH/PI mental health; serving as an important but overlooked contributor to NH/PI mental health disparities. Current findings fill critical gaps in our knowledge of NH/PI trauma and mental health while revealing the importance of screening and treating NH/PIs for trauma exposure to alleviate existing mental health disparities.

## Introduction

Paralleling other Indigenous-colonized populations, Native Hawaiians/Pacific Islanders (NH/PI) represent a vulnerable racial group deeply affected by mental health disparities and enhanced trauma risk. In limited health data, NH/PIs report among the highest rates of mental and substance use disorders of any U.S. racial group (Kaholokula, 2007; Sakai et al., 2010; Subica & Wu, 2018; Wu et al., 2013). Further, NH/PIs endure elevated rates of homicide, physical violence, and interpersonal violence (Mayeda et al., 2006; McCubbin, 2003; Ramisetty-Mikler et al., 2006). Yet, despite composing one of only six federally recognized U.S. racial groups, NH/PIs remain severely understudied and have received minimal research and clinical attention; rendering their mental health and substance use disparities – including the factors that influence them – poorly understood. In particular, almost nothing is known about the scope and impact of trauma in NH/PI populations (Archambeau et al., 2010) despite community reports suggesting trauma may be pervasive in NH/PI communities.

In the literature, trauma constitutes a well-established risk factor for poor mental health and substance use outcomes (Breslau et al., 2003; Elliott et al., 2014; Gibson et al., 2019; Rytwinski et al., 2013), with research linking lifetime trauma exposure to increased risk for psychiatric disorders and substance use (Alvarez-Alonso et al., 2016; Gibson et al., 2019; Libby et al., 2005; Mueser et al., 2002; Najavits et al., 2017). One explanation for this relationship is the diathesis-stress model, which posits that mental disorders are triggered by an interaction between a person’s pre-existing biological vulnerability toward mental illness and stress exposure in the environment. Evidence supporting the ‘self-medication hypothesis’ additionally suggests that trauma-exposed individuals may use substances to cope with or anesthetize the negative cognitions, affective states, and neurobiological pathologies triggered by trauma (Khantzian, 1997; Schimmenti et al., 2022), leading to worsened substance use, substance use disorders, and personal and community harms (Cicchetti & Handley, 2019; Leza et al., 2021).

For NH/PIs, trauma exposure poses a grave yet unexplored mental health concern as NH/PIs have suffered extensive cultural trauma at the hands of the U.S. (e.g. military-backed overthrow of the Hawaiian monarchy, extensive nuclear testing in the Marshall Islands), which may promote trauma risk and contribute to health disparities in present day NH/PI populations (McElfish et al., 2015; Yamada, 2004). These concerns are supported by studies suggesting that trauma exposure may be especially common and contribute to negative mental health and substance use outcomes in racial groups that have endured extensive cultural trauma (e.g. physical/cultural genocide, forcible relocation, and slavery; Brave Heart et al., 2016; O’Connell et al., 2005). For instance, research has suggested that American Indian’s legacies of cultural trauma may continue to exert intergenerational effects by generating adverse environments/contexts (Ka’apu & Burnette, 2019) that lead to elevated rates of trauma exposure, mental health difficulties, and substance use within American Indian communities (Beals et al., 2013; John-Henderson & Ginty, 2020; Manson et al., 2005; Robin et al., 1996, 1997; Skewes & Blume, 2019).

Unfortunately, while strong literature exists regarding trauma’s deleterious effects on other U.S. minority and Indigenous-colonized populations, almost no known studies have investigated trauma and its mental health consequences in NH/PIs (Archambeau et al., 2010). This lack of research presents an ethical and pragmatic concern as (1) researchers, state/federal policy makers, and community stakeholders have repeatedly highlighted the importance/need to conduct disaggregated health and mental health research with NH/PI populations (Ka’apu & Burnette, 2019; Morey et al., 2022; Shimkhada et al., 2021), and (2) a corpus of literature has revealed that NH/PIs suffer sizable mental health disparities (Subica et al., 2019a, 2020a; Subica & Wu, 2018) that may be worsened by trauma exposure, particularly during COVID-19 (Subica et al., 2023). For example, the extreme rates of death and loss endured by many NH/PI families during the COVID-19 pandemic – as NH/PIs suffered among the highest per capita COVID-19 death rates in the U.S. (Penaia et al., 2021) – may have had profound traumatic effects on NH/PI communities (Subica et al., 2022; Zagorski, 2020).

With regard to mental health, community studies conducted before COVID-19 found high rates of depression, anxiety, and alcohol disorders in NH/PI communities with 21%, 12%, and 22% of NH/PI adults experiencing major depressive disorder (MDD), generalized anxiety disorder (GAD), and alcohol use disorder (AUD), respectively: two to four times the general population rate (Subica et al., 2019b). Unfortunately, during COVID-19, adult rates of MDD, GAD, and AUD further increased to 27%, 19%, and 27%, respectively; a stark increase in behavioral health difficulties affecting NH/PI communities (Subica et al., 2023).

Regarding substance use, NH/PIs demonstrate elevated prevalence of hazardous drinking and alcohol-related harms (Subica et al., 2020b, 2022; Subica & Wu, 2018) as well as heightened rates of tobacco use for both combustible cigarettes and e-cigarettes (Lew & Tanjasiri, 2003; Mukherjea et al., 2014; Seto et al., 2016; Wills et al., 2017); a major public health concern as tobacco-related diseases compose the leading causes of NH/PI morbidity and mortality (Centers for Disease Control and Prevention [CDC], 2019a, 2019b; Martin et al., 2013). Notably, community studies have found NH/PI adult smoking rates of up to 31% (including 52% for NH/PI young adults) (Caraballo et al., 2008; Mukherjea et al., 2014; Subica et al., 2020b) with a recent multi-state study revealing 22% of NH/PI adults currently smoke (Subica et al., 2023) versus 12% of the U.S. population (CDC, 2022). Similarly, 17% of NH/PI adults report lifetime e-cigarette use with 39% of NH/PI young adults reporting current e-cigarette use (Subica et al., 2020b, 2023), exceeding e-cigarette use rates for the U.S. population. Lastly, over one third of NH/PI adults report engaging in lifetime illicit substance use (e.g. cannabis, illicit opioid, and methamphetamine use); a critical area of investigation for this study as (1) lifetime illicit substance use is strongly associated with increased addiction and harm (Degenhardt & Hall, 2012), and (2) opioids and methamphetamines comprise the primary drivers of the U.S. drug overdose epidemic that consumes over 100,000 lives annually (Spencer et al., 2023).

Due to experiencing substantial disparities in mental illness and substance use, NH/PIs also possess high need and unmet need for formal mental health treatment as 33% to 35% of NH/PI adults report needing past-year mental health treatment with 60% to 76% delaying or avoiding needed treatment across studies (Subica et al., 2019a, 2023). Thus, as NH/PIs rarely seek or receive treatment for reasons that include strong mental illness stigma, lack of culturally responsive services/interventions, and unfamiliarity with the treatment process (Subica et al., 2019a; Subica & Brown, 2020), reducing treatment disparities in NH/PI populations may require identifying the core underlying factors such as trauma that contribute to NH/PIs’ concerning treatment need.

### Purpose

This novel investigation sought to close the empirical gap in NH/PI trauma and mental health research by examining the previously unstudied prevalence and impact of trauma on NH/PI mental health, substance use, and treatment need. Funded by the U.S. National Institute of Mental Health, this study capitalized on established academic-community partnerships to collect original data to guide clinicians and researchers in understanding/intervening on the diverse mental health and substance use challenges facing NH/PI communities. Based on community reports and extant research with non-NH/PI populations, we hypothesized that NH/PI trauma exposure would be associated with increased mental health symptomology, substance use, and treatment need, independent of key demographic and mental health risk variables. Informed by the principles of health equity and inclusivity, at our community partners’ requests we also included non-binary NH/PIs – who are historically viewed as culturally significant in many NH/PI societies (Semenyna & Vasey, 2019; Stip, 2015; Vasey & Bartlett, 2007) – as prior research indicated that non-binary NH/PIs may also bear significant mental health risk (Subica et al., 2023).

## Methods

### Study sample and data collection

The *Institutional Review Board of the University of California, Riverside approved all research protocols (Approval No. HS-19-310). Informed consent was obtained from all participants*. Following best practices for recruiting hard-to-reach populations, trained NH/PI-heritage staff from our community partners recruited 306 NH/PI adults (18 years and older) who originally hailed from U.S. states with significant NH/PI populations or Pacific nations. These included Hawai‘i, California, Washington, Nevada, American Samoa, Tonga, and Guam.

Recruitment occurred via purposive sampling – specifically systematic nonprobabilistic sampling (Mays & Pope, 1995) to deliberately sample NH/PI adults from multiple genders and a wide range of ages and locations to capture a broad range of participant experiences across diverse NH/PI communities. Trained staff conducted stratified multi-site recruitment by conducting data collection through three channels: in person paper-and-pencil surveys administered at NH/PI events, organizations, and locations where NH/PIs congregate (e.g. sports parks and churches), via interview format over the telephone, and self-report online surveys through Qualtrics.

### Measures

#### Demographics

Demographics included age, gender (female, male, non-binary), income, and marital status.

#### Mental health, alcohol/substance use, and treatment need

Depression and anxiety symptomology were assessed via the 9-item Patient Health Questionnaire (PHQ-9; Kroenke et al., 2001) and 7-item Generalized Anxiety Disorder scale (GAD-7; Spitzer et al., 2006), respectively. Both measures are cumulatively scored with higher scores indicating greater levels of depression or anxiety symptomology, and both utilize a 10-point diagnostic cut-point indicating MDD and GAD, respectively (Kroenke et al., 2001; Spitzer et al., 2006). Current alcohol use and probable AUD were assessed via the 3-item Alcohol Use Disorders Identification Test-Consumption (AUDIT-C; Bush et al., 1998). Higher cumulative scores represent higher levels of hazardous alcohol use with cut-off scores of ⩾4 for men and non-binary individuals and ⩾3 for women indicating probable AUD (Bush et al., 1998; Dawson et al., 2012). Internal consistency of the PHQ-9, GAD-7, and AUDIT-C were sound with Cronbach’s α of .90, .93, and .78, respectively.

Lifetime cigarette and e-cigarette were assessed using the items: ‘*Have you smoked at least 100 cigarettes in your entire life?*’ and ‘*Have you ever used any type of e-cigarette or similar vaping device?’* Current cigarette and e-cigarette use were assessed via: ‘*Do you smoke cigarettes every day, some days, or not at all?*’ and ‘*How often, if at all, do you currently use an e-cigarette or similar vaping device?*’

Lifetime illicit substance use was defined as any lifetime use of cannabis, prescription opioid, heroin, or methamphetamine use and were assessed with the items: ‘*Have you ever, even once, tried marijuana or hashish in any form*?’. ‘*Have you ever taken prescription pain pills other than ordered by your doctor?*’. ‘*Have you ever taken heroin?*’. ‘*Have you ever used methamphetamines?*’ We targeted these substances as cannabis is the most commonly used illicit substance by NH/PIs (Subica et al., 2022) while opioids and methamphetamines are the illicit substances of gravest clinical/community concern as (1) opioids are responsible for over 150 U.S. drug overdose deaths per day, and (2) the methamphetamine-caused overdose U.S. death rate has more than quadrupled from 2016 to 2021 (Spencer et al., 2023).

Need for, and delay or avoidance of formal mental health treatment were assessed using two items from the Medical Expenditure Panel Survey (AHRQ, n.d.) that prior studies have successfully used to assess NH/PI treatment need (Subica et al., 2019b, 2022): ‘*In the past 12 months during the pandemic, was there a time when you wanted to talk with or seek help about stress, depression, or problems with emotions?*’ and ‘*Did you delay or not get the care you thought you needed?*’

#### Trauma exposure

Lifetime history of traumatic exposure was assessed via the Trauma Assessment for Adults – Brief Revised Version (TAA-R; Cusack et al., 2004), a validated dichotomous measure that assesses exposure to 12 distinct types of traumatic events (e.g. childhood physical abuse, forced sexual assault, and serious accident). Summary scores for the TAA-R indicate the number of distinct traumatic event types experienced by participants.

### Statistical analyses

Analyses were performed in SPSS v.28. Descriptive statistics of frequencies, means, and standard deviations were calculated. Chi-square and one-way ANOVA tests examined gender differences in trauma exposure. Linear and logistic regressions were conducted to explore if trauma exposure significantly associated with study mental health and substance use variables after accounting for relevant demographic and mental health variables. Specifically, three linear regression models were tested with outcome variables of depression, anxiety, and alcohol use symptomology and predictor variables of (1) demographics of age, gender, income, marital status, (2) screened MDD, GAD, and AUD, and (3) number of traumatic event types experienced. Using the same predictor variables, four logistic regression models were also analyzed with outcome variables of mental health treatment need, lifetime illicit substance use, current alcohol use, and current cigarette use.

## Results

### Sample characteristics

Table 1 presents sample characteristics including frequencies and means for study mental health, substance use, and trauma variables. The mean sample age was 35 years with 51% of participants self-identifying as women, 35% as men, and 14% as non-binary gender. Screened prevalence for MDD, GAD, and AUD were high with 27%, 21%, and 36% of participants meeting diagnostic thresholds for these disorders, respectively. Nearly half of participants reported needing past-year mental health treatment (47%) with 79% of participants who reported needing treatment avoiding or delaying formal treatment.

**Table 1.** Sample characteristics and descriptive statistics.

Variables	Total sample n = 306		Women n = 157		Men *n* = 105		Non-binary gender *n* = 44	
	%	*n*	%	*n*	%	*n*	%	*n*
Income								
<20,000	13	39	13	20	12	13	14	6
$20,000–$59,999	36	111	42	66	26	27	41	18
$60,000–$100,000+	37	113	34	53	45	47	30	13
Prefer not to answer	14	43	12	18	17	18	16	7
Marital status								
Single/separated/divorced	56	171	49	77	50	52	96	42
Married/living as married	38	116	43	67	46	48	2	1
Separated/divorced	6	19	8	13	4	5	2	1
Major depressive disorder	27	83	29	45	23	24	32	14
Generalized anxiety disorder	21	64	22	34	18	19	25	11
Alcohol use disorder	36	110	26	41	47	49	46	20
Lifetime cigarette use	34	103	31	48	38	40	34	15
Current cigarette use	29	90	22	34	35	37	43	19
Lifetime e-cigarette use	38	117	36	56	38	40	48	21
Current e-cigarette use	28	85	26	41	27	28	36	16
Lifetime illicit substance use	60	183	54	84	56	59	91	40
Lifetime cannabis use	58	177	51	80	54	57	91	40
Lifetime illicit rx opioids	14	42	8	12	14	15	34	15
Lifetime heroin	2	6	1	2	3	3	2	1
Lifetime methamphetamines	6	19	5	8	9	9	5	2
MH treatment need (past year)	47	145	43	68	46	48	66	29
Avoid/delayed MH treatment	37	114	33	52	37	39	52	23
	*M*	*SD*	*M*	*SD*	*M*	i	*M*	*SD*
Age, years	35.20	11.57	35.21	11.67	36.54	12.42	32.00	8.19
Trauma (TAA)^a^	2.53	2.65	2.24	2.48	2.45	2.75	3.75	2.68
Depression (PHQ-9)^b^	6.89	5.89	7.11	6.19	6.19	5.40	7.75	5.90
Anxiety (GAD-7)^c^	5.39	5.25	5.41	5.49	5.24	4.87	5.68	5.37
Alcohol (AUDIT-C)^d^	2.74	2.58	2.14	2.19	3.36	2.91	3.41	2.57

*Note*. Rx, prescription. Lifetime illicit substance use refers to any lifetime cannabis, prescription opioid, heroin, or methamphetamine use.

a Scoring range on the Trauma Assessment for Adults scale is 0 to 13. Higher scores indicate greater number of traumatic event types experienced.

b Scoring range on the Patient Health Questionnaire-9 (PHQ-9) is 0 to 27. Higher scores indicate greater depressive severity.

c Scoring range on the Generalized Anxiety Disorder-7 (GAD-7) is 0 to 21. Higher scores indicating greater anxiety severity.

d Scoring range on the Alcohol Use Disorders Identification Test-Concise (AUDIT-C) is 0 to 12. Higher scores indicate greater hazardous alcohol use.

Lifetime trauma prevalence among NH/PI participants was 69%. On average, participants reported lifetime exposure to 2.5 distinct types of traumatic events. Women and men did not significantly differ in their overall mean number of distinct traumatic event types experienced while non-binary participants had significantly higher mean traumatic event type exposure scores than both women (*p* < .01) and men (*p* < .05).

Detailed in Table 2, the most prevalent traumatic events among participants were childhood physical abuse (34%), witnessing someone seriously injured or killed (29%), forced sexual assault at any age (27%), childhood sexual abuse before age 18 years (25%), and having a close friend or family member killed or murdered by another person (23%). NH/PI women and men showed no significant differences in experiencing any of the assessed traumatic events in their lifetime. By contrast, non-binary participants reported significantly higher prevalence (*p* < .05) than (1) women of experiencing childhood sexual abuse before age 13 and 18 years, forced sexual assault, physical assault with a weapon, and a serious accident, and (2) men of experiencing childhood sexual abuse before age 13 and 18 years, and forced sexual assault.

**Table 2.** Prevalence of traumatic events by gender.

	Total sample		Women		Men		Non-binary gender	
	*n* *=* 306		*n* = 157		*n* = 105		*n* = 44	
	%	*n*	%	*N*	%	*n*	%	*n*
Traumatic event type								
Child physical abuse	34	105	33	52	31	33	46	20
Child sexual abuse (>age 13 years)	20	61	15	23	20	21	39	17
Child sexual abuse (>age 18 years)	25	75	22	35	21	22	41	18
Forced sexual assault	27	81	25	39	22	23	43	19
Physical assault with weapon	16	50	12	18	19	20	27	12
Physical assault no weapon	13	38	10	16	14	15	16	7
Serious accident	18	55	14	21	20	21	30	13
Saw serious violence/injury	29	87	23	36	33	35	36	16
Natural disaster	22	68	19	30	22	23	34	15
Serious illness	5	15	3	5	8	8	5	2
Military/combat experience	2	6	3	4	1	1	2	1
Close friend/family killed	23	70	22	34	23	24	27	12
Other	21	64	24	37	13	14	30	13

### Linear and logistic regressions

Linear regressions were performed to determine if trauma exposure significantly associated with increased NH/PI depression and anxiety symptomology, and hazardous alcohol use (Table 3). For all three regression models, no demographic variables significantly associated with the outcome variables. Supporting study hypotheses, exposure to multiple traumatic event types independently associated (*p* < .01) with increased depression symptomology (β = .19), anxiety symptomology (β = .19), and hazardous alcohol use (β = .30), even after accounting for demographics and potential confounding mental health risk factors (e.g. GAD and AUD for the depression symptomology model).

**Table 3.** Linear regressions by demographics, major depressive/generalized anxiety/alcohol use disorders, and trauma exposure.

Variables	β	*b* (*SE*)	*F*	*R*^2^ (*∆R*^2^)
DV = depression symptomology				
Age	.02	0.01 (.02)	3.56**	.08**
Gender (reference: Female)				
Male	−.08	−.93 (.54)		
Non-binary	−.04	−.86 (.93)		
Income (reference: <$20,000)				
$20,000–$59,999	.09	1.09 (.61)		
$60,000+	.05	.89 (.62)		
Marital status (reference: single)				
Married	−.09	−1.07 (.58)		
Separated/divorced	−.07	−1.68 (1.07)		
Generalized anxiety disorder^a^	.58**	8.42** (.63)**	32.16**	.53** (.45)**
Alcohol use disorder^b^	.15**	1.87** (.56)**		
Trauma exposure	.19**	.41** (.10)**		
DV = anxiety symptomology				
Age	−.09	−.04 (.02)	3.37**	.08**
Gender (reference: Female)				
Male	−.03	−.29 (.49)		
Non-binary	−.08	−1.48 (.84)		
Income (reference: <$20,000)				
$20,000–$59,999	.03	.31 (.55)		
$60,000+	.02	.26 (.56)		
Marital status (reference: single)				
Married	.03	.28 (.52)		
Separated/divorced	−.06	−1.20 (.97)		
Alcohol use disorder^b^	.10*	1.12* (.50)*	29.40**	.51** (.43)**
Major depressive disorder^c^	.58**	6.76** (.52)**		
Trauma exposure	.19**	.36** (.09)**		
DV = alcohol use disorder symptomology				
Age	−.06	−.01 (.01)	5.99**	.13**
Gender (reference: Female)				
Male	.25	1.34 (.29)		
Non-binary	.16	1.51 (.51)		
Income (reference: <$20,000)				
$20,000–$59,999	.10	.55 (.34)		
$60,000+	.14	.75 (.34)		
Marital status (reference: single)				
Married	−.12	−.66 (.32)		
Separated/divorced	−.05	−.59 (.59)		
Generalized anxiety disorder^a^	.14*	.89* (.41)*	9.69**	.25** (.12)**
Major depressive disorder^c^	.03	.18 (.39)		
Trauma exposure	.30**	.30** (.05)**		

*Note*. DV = dependent variable; β = standardized regression coefficient; *b* = unstandardized regression coefficient; *SE* = standard error; *R*^2^ = coefficient of determination; *∆R*^2^ = change in coefficient of determination.

a Determined via Generalized Anxiety Disorder-7.

b Determined via Alcohol Use Disorders Identification Test-Consumption.

c Determined via Patient Health Questionnaire-9.

* *p* *<* .05. **p *<* .01.

Logistic regression results are displayed in Table 4. For mental health treatment need, each 1-point increase in traumatic event type experienced independently associated with 1.4 times greater adjusted odds of needing treatment (*p* < .01), after accounting for demographic variables and the effects of MDD, GAD, and AUD. Similarly, for the substance use models, each 1-point increase in traumatic event type experienced independently associated with 1.6, 1.4, and 1.1 times greater adjusted odds of engaging in lifetime illicit substance use, current alcohol use, and current cigarette use (*p* < .01), respectively.

**Table 4.** Logistic regressions by demographics, major depressive/generalized anxiety/alcohol use disorders, and trauma exposure.

Variables	AOR	95% CI
DV = Mental health treatment need		
Age in years	.98	[0.95, 1.01]
Gender (reference: female)		
Male	1.32	[0.71, 2.48]
Non-binary	2.24	[0.83, 6.04]
Income (reference: <$20,000)		
$20,000–$59,999	1.93	[0.95, 3.94]
$60,000–$100,000+	2.42*	[1.19, 4.92*]
Marital status (reference: single)		
Married	1.09	[0.56, 2.10]
Separated/divorced	.80	[0.22, 2.93]
Major depressive disorder^a^	7.13*	[3.17, 16.01*]
Generalized anxiety disorder^b^	1.40	[0.57, 3.45]
Alcohol use disorder^c^	.83	[0.44, 1.57]
Trauma^d^	1.36**	[1.21, 1.54**]
DV = Illicit substance use		
Age in years	.98	[0.96, 1.01]
Gender (reference: female)		
Male	.78	[0.42, 1.44]
Non-binary	4.47*	[1.11, 18.19*]
Income (reference: <$20,000)		
$20,000–$59,999	1.61	[0.79, 3.27]
$60,000–$100,000+	1.94	[0.95, 3.98]
Marital status (reference: single)		
Married	1.15	[0.60, 2.21]
Separated/divorced	.54	[0.14, 2.02]
Major depressive disorder^a^	.99	[0.44, 2.23]
Generalized anxiety disorder^b^	.58	[0.24, 1.39]
Alcohol use disorder^c^	1.96*	[1.01, 3.80*]
Trauma^d^	1.63**	[1.40, 1.90**]
DV = Current alcohol use		
Age in years	.99	[0.97, 1.02]
Gender (reference: female)		
Male	1.08	[0.59, 1.97]
Non-binary	1.44	[0.45, 4.58]
Income (reference: <$20,000)		
$20,000–$59,999	1.78	[0.92, 3.45]
$60,000–$100,000+	4.44**	[2.13, 9.26**]
Marital status (reference: single)		
Married	.50*	[0.26, 0.96*]
Separated/divorced	.51	[0.15, 1.78]
Major depressive disorder^a^	.67	[0.30, 1.47]
Generalized anxiety disorder^b^	1.92	[0.78, 4.72]
Trauma^d^	1.44**	[1.24, 1.67**]
DV = Current smoking		
Age in years	1.01	[0.98, 1.03]
Gender (reference: female)		
Male	1.45	[0.80, 2.62]
Non-binary	2.00	[0.76, 5.26]
Income (reference: <$20,000)		
$20,000–$59,999	.87	[0.45, 1.70]
$60,000–$100,000+	.68	[0.34, 1.34]
Marital status (reference: single)		
Married	1.16	[0.61, 2.22]
Separated/divorced	.88	[0.26, 2.96]
Major depressive disorder^a^	1.45	[0.70, 2.97]
Generalized anxiety disorder^b^	.84	[0.38, 1.85]
Alcohol use disorder^c^	2.29*	[1.27, 4.13*]
Trauma^d^	1.14*	[1.03, 1.27*]

*Note*. AOR = adjusted odds ratio; CI = confidence interval; DV = dependent variable.

a Determined via Patient Health Questionnaire-9.

b Determined via Generalized Anxiety Disorder-7.

c Determined via Alcohol Use Disorders Identification Test-Consumption.

d Determined via Trauma Assessment for Adults.

* *p* *<* .05. **p *<* .01.

## Discussion

Findings revealed that community-dwelling NH/PIs suffer extensive trauma, enduring on average two to three different types of traumatic events by the (mean) age of 35 years. Confirming community reports, trauma was pervasive in NH/PI communities with nearly 7 out of 10 NH/PI adults experiencing at least one traumatic event in their lifetime; matching reported trauma rates of 62% to 70% for Indigenous American Indian communities (Beals et al., 2013; Manson et al., 2005). Further mirroring American Indian populations (Manson et al., 2005), NH/PI women and men showed no gender differences in overall trauma exposure, diverging from the U.S. general population. In this way, the trauma patterns of NH/PIs appear to align with the trauma experiences of other Indigenous U.S. populations; potentially indicating a similar need to design culturally targeted mental health interventions specifically attuned to NH/PIs’ considerable trauma experiences.

Among NH/PIs, the most common traumatic events were childhood physical abuse, childhood sexual abuse, forced sexual assault, witnessing serious violence/injury to another person, and having a close friend or family member intentionally murdered or killed. Notably, NH/PIs’ rates of childhood abuse substantially exceeded U.S. population rates with NH/PI women’s and men’s childhood sexual abuse rates of 22% and 21%, respectively, greatly surpassing the 14% and 6% sexual abuse rates for U.S. women and men, respectively (Finkelhor et al., 2015). Similarly, NH/PIs’ 34% overall rate of childhood physical abuse substantially exceeded the 18% U.S. overall rate of childhood physical abuse (Finkelhor et al., 2015; Stoltenborgh et al., 2015), capturing for the first time the stark disparities in childhood abuse afflicting NH/PI populations. In addition, female and male NH/PIs reported equivalent rates of childhood physical and sexual abuse and lifetime forced sexual assault – an unexpected finding given that U.S. women and men typically display pronounced gender differences for these traumas with sexual abuse/violence more commonly experienced by women and physical abuse/violence more commonly experienced by men (Finkelhor et al., 2015; Stoltenborgh et al., 2015). Accordingly, clinicians and treatment settings should consider screening both NH/PI men and women for physical and sexual trauma across the lifespan as these major stressors have been shown to be critical precursors for mood, psychotic, stress, and substance use disorders (Cicchetti & Handley, 2019; Libby et al., 2005; McCall-Hosenfeld et al., 2014; Subica, 2013).

Confirming study hypotheses, experiencing more traumatic event types was associated with multiple negative mental health and substance use outcomes, even after controlling for the effects of other significant mental health risk factors (e.g. clinical depression, anxiety, and alcohol use). Specifically, greater exposure to multiple traumatic event types across the lifespan independently predicted (1) greater severity of current NH/PI depression, anxiety, and hazardous alcohol use, and (2) greater likelihood for needing formal mental health treatment – with almost one half of all NH/PI participants reporting a need for past-year treatment. These findings align with prior research with other populations indicating that trauma exposure is strongly associated with increased prevalence of depression and anxiety (Libby et al., 2005; Mandelli et al., 2015; Shalev et al., 1998). For example, large-scale reviews and meta-analytic studies examining general population data have shown that trauma-exposed children and adolescents have 2.6 times greater odds of depression than non-exposed counterparts (Vibhakar et al., 2019) while adults exposed to childhood traumas have 2.0 to 2.8 times greater odds for depression (Mandelli et al., 2015) and are 1.9 to 3.6 times more likely to develop anxiety disorders versus non-exposed adults (Fernandes & Osório, 2015). Additionally, our finding that exposure to one traumatic event type is associated with 40% greater odds of needing past-year mental health treatment in NH/PIs coheres with research in other populations (e.g. military personnel, sexual assault survivors, and primary care patients) that have linked trauma exposure to greater mental health treatment need and visits (Hidalgo & Davidson, 2000; Kartha et al., 2008; Suffoletta-Maierle et al., 2003). Importantly, prior research with non-NH/PI populations also suggests that exposure to trauma is strongly associated with reluctance to seek treatment (Kantor et al., 2017). Thus, it is possible that the high prevalence of trauma in NH/PIs found in this study may also contribute to NH/PI’s high rates of treatment avoidance found in prior NH/PI community studies (Lim et al., 2019; Subica et al., 2019a, 2023) – including an startling 79% rate of avoiding/delaying treatment found in the current NH/PI sample.

Exposure to multiple traumatic event types also predicted increased likelihood for currently using alcohol and cigarettes, and lifetime use of illicit substances – three classes of substances that induce serious health-related harms (e.g. injury, suicide, cardiovascular disease, cancer, and fatal overdose), including among NH/PIs (Lew & Tanjasiri, 2003; Subica et al., 2020a; Subica, Sampaga, et al., 2023; Tanjasiri & Peters, 2010). Overall, study data indicating that lifetime traumatization is pervasive in NH/PI communities and significantly impacts NH/PI adults’ ongoing mental health and substance use behaviors suggests that proper assessment and treatment of trauma in NH/PI populations may improve their mental health by mitigating trauma-induced consequences.

As trauma and mental health research on NH/PIs remain scarce, our data highlights the pressing need for additional research to identify, understand, and ultimately intervene on the mechanisms through which trauma infiltrates NH/PI communities and may perpetuate mental health disparities. One area of this research could involve exploring the intersection between NH/PIs’ interpersonal and cultural trauma experiences, which may have both intergenerational and contemporaneous effects in aggravating trauma, mental health, and substance use in NH/PI communities (Skewes & Blume, 2019; Subica & Link, 2022). Conducting this research will require refining and validating tailored measures for assessing cultural trauma in NH/PI populations, some of which have been recently developed (Kaholokula et al., 2010; Pokhrel & Herzog, 2014), as similar trauma measures with other Indigenous and minority populations (Armenta et al., 2016; Whitbeck et al., 2004; Williams et al., 2022) may not generalize to the unique trauma experiences of NH/PIs. Second, current NH/PI populations experience sizable social and economic disadvantage and structural racism (Kaholokula et al., 2020; Morey et al., 2022; Subica & Link, 2022); critical risk factors for trauma exposure and mental health disparities. As a result, many NH/PIs may live in high-risk environments/communities that increase their likelihood for experiencing lifetime violence, abuse, and other traumatic events, as well as serious mental health and substance use disorders (Beals et al., 2013; Ehlers et al., 2013; Evans-Campbell, 2008). Accordingly, researchers should seek to investigate the relationships between community NH/PIs’ trauma and mental health with structural inequities in the social/built environment to identify the underlying social determinants that may exacerbate trauma risk in NH/PI communities.

As an initial community study of NH/PI trauma and mental health disparities, several study limitations exist. First, data were cross-sectional, which precluded establishing causal relationships between our trauma and mental health variables. Second, this study specifically assessed traumatic event types and not frequency of traumatic experiences. Thus, our results likely underestimated the true levels of trauma exposure experienced by NH/PI participants, and trauma’s effects on NH/PI mental health and substance use, as many traumatic event types can be chronic/repeatedly experienced (e.g. childhood physical and sexual abuse, sexual assault, and combat trauma), influencing mental health in a dose-response relationship (Pérez-Fuentes et al., 2013). Finally, as this study focused on initially capturing the prevalence and impact of trauma exposure on NH/PI mental health, we did not examine the effects of post-traumatic stress on study variables and relationships; a gap that future research should address.

## Conclusion

Collectively, findings of this novel investigation indicate that trauma is a severe problem affecting NH/PI communities that may exert pernicious effects on NH/PI mental health and substance use. In this way, trauma may be linked to the expression of health inequities in NH/PI populations, constituting an important but previously unrecognized contributor for NH/PI mental health disparities. Consequently, our data strongly reinforces the need for researchers and clinicians to study, screen, and address trauma and its sequelae in NH/PI populations as it may offer an effective pathway for alleviating mental health and substance use disparities in many NH/PI communities.
